# Absorption Characteristics of Vertebrate Non-Visual Opsin, Opn3

**DOI:** 10.1371/journal.pone.0161215

**Published:** 2016-08-17

**Authors:** Tomohiro Sugihara, Takashi Nagata, Benjamin Mason, Mitsumasa Koyanagi, Akihisa Terakita

**Affiliations:** 1 Department of Biology and Geosciences, Graduate School of Science, Osaka City University, Sugimoto, Sumiyoshi-ku, Osaka, Japan; 2 Department of Genetics, Stanford School of Medicine, Stanford University, Stanford, California, United States of America; 3 Japan Science and Technology Agency (JST), Precursory Research for Embryonic Science and Technology (PRESTO), Kawaguchi, Saitama, Japan; University of Western Australia, AUSTRALIA

## Abstract

Most animals possess multiple opsins which sense light for visual and non-visual functions. Here, we show spectral characteristics of non-visual opsins, vertebrate Opn3s, which are widely distributed among vertebrates. We successfully expressed zebrafish Opn3 in mammalian cultured cells and measured its absorption spectrum spectroscopically. When incubated with 11-*cis* retinal, zebrafish Opn3 formed a blue-sensitive photopigment with an absorption maximum around 465 nm. The Opn3 converts to an all-*trans* retinal-bearing photoproduct with an absorption spectrum similar to the dark state following brief blue-light irradiation. The photoproduct experienced a remarkable blue-shift, with changes in position of the isosbestic point, during further irradiation. We then used a cAMP-dependent luciferase reporter assay to investigate light-dependent cAMP responses in cultured cells expressing zebrafish, pufferfish, anole and chicken Opn3. The wild type opsins did not produce responses, but cells expressing chimera mutants (WT Opn3s in which the third intracellular loops were replaced with the third intracellular loop of a Gs-coupled jellyfish opsin) displayed light-dependent changes in cAMP. The results suggest that Opn3 is capable of activating G protein(s) in a light-dependent manner. Finally, we used this assay to measure the relative wavelength-dependent response of cells expressing Opn3 chimeras to multiple quantally-matched stimuli. The inferred spectral sensitivity curve of zebrafish Opn3 accurately matched the measured absorption spectrum. We were unable to estimate the spectral sensitivity curve of mouse or anole Opn3, but, like zebrafish Opn3, the chicken and pufferfish Opn3-JiL3 chimeras also formed blue-sensitive pigments. These findings suggest that vertebrate Opn3s may form blue-sensitive G protein-coupled pigments. Further, we suggest that the method described here, combining a cAMP-dependent luciferase reporter assay with chimeric opsins possessing the third intracellular loop of jellyfish opsin, is a versatile approach for estimating absorption spectra of opsins with unknown signaling cascades or for which absorption spectra are difficult to obtain.

## Introduction

Most animals capture light with opsins for vision and non-visual functions. Opsins form light-sensitive pigments with a retinal chromophore and absorb light to activate G proteins. Therefore, opsins are considered to be light-sensitive G protein coupled receptors (GPCRs). Several thousands of opsin genes have been identified, and they are phylogenetically and functionally classified into eight groups [1–3]. Accumulated evidence has revealed that six of the groups (the Gt-coupled opsin, Opn3, Gq-coupled opsin, Go-coupled opsin, Gs-coupled opsin and Opn5 groups) contain G-protein-coupled opsins [4–12], although the members of the other two groups, retinochromes and peropsins, serve as retinal photoisomerases [13–16]. Most G-protein coupled opsin groups are composed of some vertebrate subgroups in addition to invertebrate homologs, and functional differences among the subgroups is an important issue to be solved for complete understanding of vertebrate opsin diversity. In addition, the fact that mammals possess opsins of limited subgroups, compared to non-mammalian vertebrates, in most opsin groups indicates that functional properties of opsins in such subgroups could provide a clue to an overall understanding of the mammalian photoreception [17–19].

The Opn3 group, which is closely related to the Gt-coupled opsin group, contains the vertebrate Opn3 subgroup including mammalian Opn3 (originally called encephalopsin or panopsin) and three TMT (teleost multiple tissue) opsin subgroups in addition to the invertebrate Opn3 homologs (invertebrate c-opsins or pteropsins from organisms such as amphioxus, insects and annelids) [5, 20]. Interestingly, the members of the vertebrate Opn3 and TMT opsin subgroups are expressed in various vertebrate tissues, including the brain (human, mouse, pufferfish, and zebrafish), liver (human and pufferfish), kidney (human and zebrafish), and heart (zebrafish), in addition to the eye (human, pufferfish, and zebrafish) [21–25]. The mRNA localizations of the vertebrate Opn3 and TMT opsins suggest that the members of the Opn3 group may confer photosensitivity in extraocular tissues that are generally considered light-insensitive in vertebrates, if they indeed serve as light-sensor proteins.

Recently, we attempted to analyze the vertebrate opsins in the Opn3 group, and we successfully expressed the pufferfish TMT opsin and reported its molecular properties [5]. The pufferfish TMT opsin has an absorption maximum in the blue region and activates Gi-type and Go-type G proteins in a light-dependent manner, indicating that it potentially serves as a light-sensitive Gi/Go-coupled receptor [5]. Medaka TMT opsins were also shown to be blue-sensitive Gi/Go coupled opsins [26]. Although absorption spectra of the vertebrate Opn3 have not been obtained, probably due to their low expression level in cultured cells, light-dependent cellular responses in cultured cells expressing medaka Opn3 were measured using an impedance-based Real-Time Cell Analysis (RTCA) system, a method for detecting cellular responses through changes in cellular morphology elicited by surface receptor activation [27]. This study suggested that vertebrate Opn3 could serve as a light-sensor protein. However, the molecular properties of vertebrate Opn3, including its absorption spectra and ability to activate G proteins, remain unsolved.

Here, we investigated spectral properties of several vertebrate Opn3s and compare them with those of TMT opsins. We show that zebrafish Opn3 formed a blue-sensitive photopigment. We then used quantified wavelength-dependent intracellular cAMP responses of cultured cells expressing zebrafish, pufferfish, and chicken Opn3 chimeras. We suggest that the vertebrate Opn3s have the ability to activate G proteins and that pufferfish and chicken Opn3s possess absorption characteristics similar to zebrafish Opn3.

## Materials and Methods

### Ethics Statement

This experiment was approved by the Osaka City University animal experiment committee (#S0032) and complied with the Regulations on Animal Experiments from Osaka City University.

### Animals

Wild-type (AB strain) zebrafish (*Danio rerio*) were obtained from the Zebrafish International Resource Center (ZIRC) and National Bio Resource Project (NBRP) Zebrafish. Pufferfish (*Takifugu rubripes*) and C57BL/6 mice (*Mus musculus*) were obtained from Shimizu Laboratory Supplies Co., Ltd. (Kyoto, Japan) and Oriental Yeast Co., Ltd. (Tokyo, Japan), respectively. Zebrafish were housed at 28.5°C under 14-h light/10-h dark cycles and were fed every day with newly hatched brine shrimp (*Artemia* nauplii). Mice were maintained at 24°C on 12-h light/12-h dark cycles and provided food and tap water *ad libitum*.

### Dissection and RNA isolation

Three adult zebrafish and an adult pufferfish were euthanized by decapitation after anesthetized with ice. Two mice (female 10-week-old) were euthanized with CO_2._ Their brains were collected and total RNAs were extracted from the brain tissues using Sepasol(R)-RNA I (Nacalai Tesque Inc., Kyoto, Japan).

### Expression vectors of opsins and their mutants

The cDNAs of the zebrafish, pufferfish, and mouse Opn3s were isolated from the total RNA of brain tissues of the respective animals by RT-PCR. The DNA of human Opn3 was obtained by combining DNA fragments amplified from the genome DNA of HEK293S cells (a human embryonic kidney cell line) by PCR. In each case, PCR was carried out using gene-specific primers based on gene sequences found in genome databases. Because we previously reported that the C-terminal truncation of long-tailed opsins increased the purification efficiency of the pigment [5, 28, 29], we constructed deletion mutants of Opn3s that have a shorter C terminus. The cDNAs of C-terminal-truncated anole Opn3 (minus the C-terminal 51 amino acids) and full-length chicken Opn3 were synthesized based on gene sequences found in genome databases (Genscript). The cDNAs of C-terminal-truncated zebrafish, pufferfish, chicken, mouse, and human Opn3s (minus the C-terminal 52, 54, 51, 51, and 51 amino acids, respectively) were generated from respective full-length cDNAs. Chimeric mutants having the third intracellular loop of Gs-coupled Jellyfish opsins (S1 Fig), which were deduced based on the previous report [30], were generated by replacing the cDNA region corresponding to the third intracellular loop of opsins with that of Gs-coupled jellyfish opsin by PCR. The C-termini of WT, C-terminal-truncated and chimera Opn3s were tagged with the rho 1D4 epitope sequence (ETSQVAPA) [31]. The tagged cDNAs were inserted between the *Hind* III and *Eco* RI sites of the pcDNA3.1 expression vector (Invitrogen). The expression constructs of pufferfish TMT opsin [5], C-terminal-truncated mosquito Opn3 [5], spider visual opsin Rh1 [32], jellyfish opsin [10], and bovine rhodopsin [33], each possessing the C-terminal 1D4 sequence, were also used.

### Expression and purification of the opsin-based pigment and spectroscopy

The opsin expression and purification were performed as described previously [34, 35]. Briefly, the opsin expression vectors were transfected into HEK293S cells or COS-1 cells (SV40-transformed African green monkey kidney cell line) using the polyethylenimine (PEI) transfection method. The transfected cells were harvested two days after the transfection. After addition of 11-*cis* retinal, opsin-based pigments were extracted with 1% dodecyl β-D-maltoside (DM) in HEPES buffer (pH 6.5) containing 140 mM NaCl and 3 mM MgCl_2_ (buffer A), bound to 1D4-agarose, washed with 0.02% DM in buffer A and eluted with buffer A containing 0.02% DM and C-terminal peptide of bovine rhodopsin as described.

The absorption spectra of the opsin-based pigments were recorded at 4°C by using a Shimadzu UV2450 spectrophotometer. Blue lights were supplied by a 1-kW halogen lamp (Philips) with a 470 nm interference filter (MZ0470; Asahi Spectra Co., Ltd., Tokyo, Japan).

### Chromophore analysis

The chromophore configurations of the irradiated and non-irradiated, purified zebrafish Opn3 were analyzed by HPLC, as described [16, 36].

### Measurement of intracellular cAMP level

The changes in the intracellular cAMP level of the opsin-expressing HEK293S cells were measured using the GloSensor cAMP assay (Promega) as described [5]. Briefly, 35mm tissue culture dishes of HEK293S cells (20–30% confluent) were transfected with 1.5 μg of each opsin expression plasmid and 1.5 μg of the pGloSensor-22F cAMP plasmid (Promega) using the PEI transfection method. The transfected cells were incubated overnight in culture medium containing 10% FBS and supplemented with 11-*cis* retinal. Before measurements, the culture medium was replaced with a CO_2_-independent medium containing 10% FBS and GloSensor cAMP Reagent stock solution (Promega). Dishes of cells were allowed to equilibrate with the recording media at 25°C for at least two hours. After equilibration, cells were then stimulated with light (see details below) and the change in luminescence, an indicator of intracellular cAMP, was measured at 25°C using a GloMax 20/20n Luminometer (Promega). To measure light-dependent Gi activation, (light-dependent cAMP decrease), the cells were first treated with 3.5 μM forskolin, a direct activator of adenylyl cyclase, to produce an increase in the intracellular cAMP level. The light-induced changes in the cAMP level in the transfected cells were measured by irradiation with a green (500 nm) light-emitting diode (LED) light (Ex-DHC; Bio Tools Inc., Gunma, Japan). The light stimuli were applied for 5 seconds in all GloSensor cAMP assays.

### Estimation of spectral sensitivity curves

LEDs with spectral emission peaks of 410 nm, 430 nm, 470 nm, 510 nm, 540nm 580 nm, 600 nm and 630 nm arrayed on a board (SPL-25-CC; REVOX Inc., Kanagawa, Japan) were used as light sources. The quantal intensities of each wavelength LED light were matched to 1.5 * 10^13^, 1.9 * 10^14^, or 1.9 * 10^15^ photons / cm^2^ · sec using interference filters (MZ0410, MZ0430, MZ0470, MZ0510, MZ0540, MZ0580, and MZ0600; Asahi Spectra Co., Ltd.), neutral-density (ND) filters (SIGMAKOKI Co., Ltd., Saitama, Japan and Shibuya Optical Co., Ltd., Saitama, Japan) and ground-glass (Shibuya Optical Co., Ltd.) (S2 Fig). The wavelength-dependent responses (change in luminescence; cAMP) of opsin-expressing cultured cells were measured for each light stimuli. The measured luminescence values were normalized to those just before the irradiations to obtain the wavelength-dependent responses. Dose (intensity)-response curves were generated for cultured cells expressing each of the opsins by irradiating cells with green (500 nm) LED light at multiple intensities, established using a series of neutral-density (ND) filters. It should be noted that individual dishes of cells were irradiated only once during the measurements and at least three independent measurements were made at each wavelength or intensity. The intensity-response curve was obtained by fitting a sigmoid function (*V* = *V*_*max*_ * *I*^*n*^ / (*I*^*n*^ + *K*^*n*^), where *V* is the value of response, *V*_*max*_ is maximum response value, *I* is the intensity of light stimulus, *K* is intensity of light stimulus eliciting 50% *V*_*max*_, and *n* is the exponent) to the mean responses at each intensity of 500 nm light. The values of the wavelength-dependent responses were extrapolated to the intensity-response curve to transform the values into photon numbers required for the responses, equivalent to the relative sensitivity [37]. Spectral sensitivity curves were estimated by fitting a rhodopsin nomogram curve [38] to the relative sensitivities according to the least squares method with the aid of IGOR Pro software (WaveMetrics).

## Results

### Absorption spectrum of zebrafish Opn3

**Fig 1:**
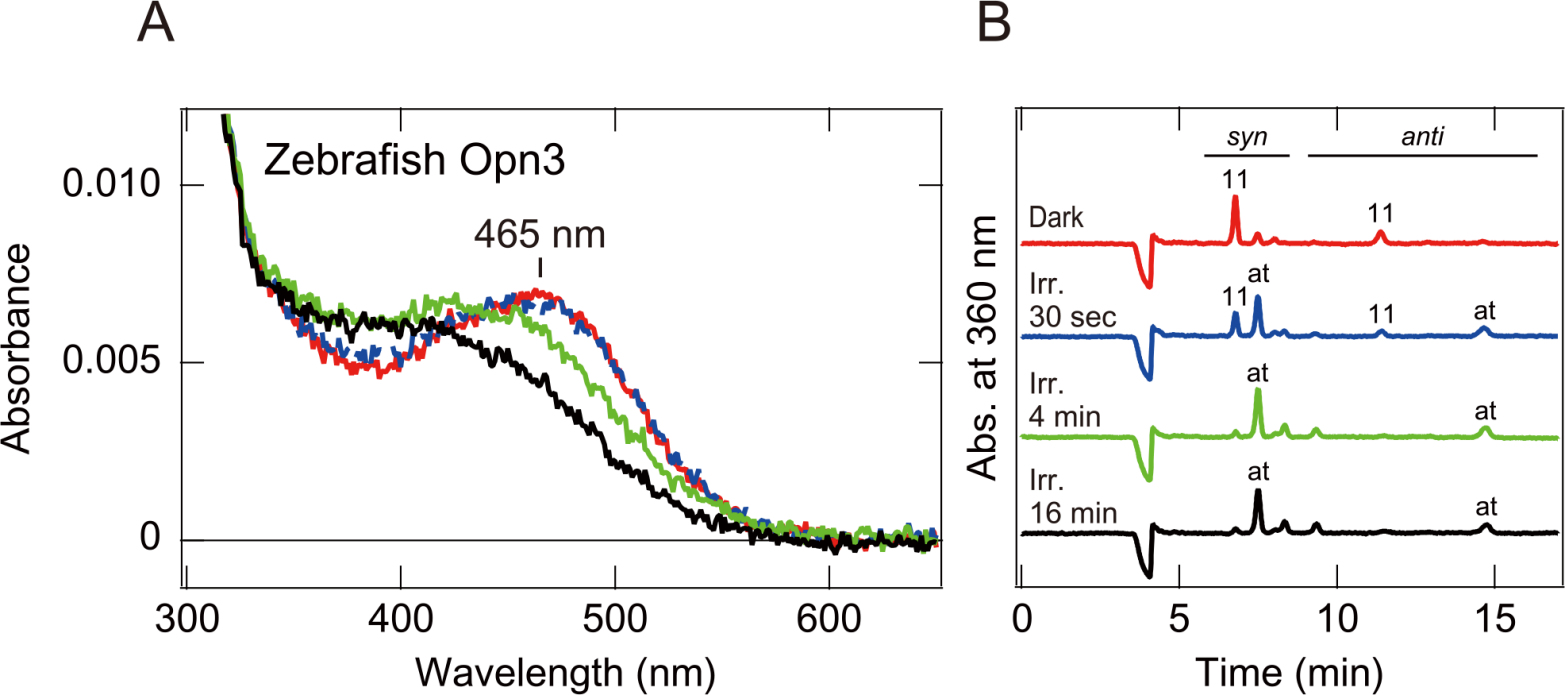
Absorption spectra and chromophore configurations of zebrafish Opn3 before and after light irradiation. (A) Absorption spectra of purified zebrafish Opn3 in the dark (red curve), after 30 seconds (blue dotted curve), 4 minutes (green curve) and 16 minutes (black curve) irradiation with blue light. (B) Chromophore configurations of purified zebrafish Opn3 in the respective states.

We first attempted to investigate the absorption spectra of several vertebrate (pufferfish, zebrafish, anole, chicken, mouse and human) Opn3s, using spectroscopic analysis of the recombinant Opn3s, expressed in HEK293 cells and purified after addition of 11-*cis* retinal. We successfully measured an absorption spectrum of zebrafish Opn3, which had an absorption maximum at around 465 nm (Fig 1A). The absorption spectrum of the zebrafish Opn3 was similar to that of the pufferfish TMT opsin that we previously reported [5]. Unfortunately, we were unable to obtain clear absorption spectra for the other Opn3s, probably due to their low expression level in cultured cells.

We then investigated spectral changes of zebrafish Opn3 upon light irradiation. Irradiation of zebrafish Opn3 with blue light for 30 sec did not cause any obvious spectral changes (Fig 1A). Chromophore analyses of zebrafish Opn3, before and after the 30 sec light-irradiation, indicated that the Opn3 bound to 11-*cis* retinal in the dark, and approximately half of the chromophore underwent photoisomerization to all-*trans* retinal during blue light irradiation (Fig 1B). These results show formation of the photoproduct with an absorption spectrum almost identical to the dark state one. Further irradiation caused an obvious blue shift of the spectrum with changes in the positions of isosbestic point, suggesting that the photoproduct was changed to a spectroscopically different product(s) possessing an all-*trans* chromophore (Fig 1A and 1B). These findings indicate that the teleost Opn3 and TMT opsin have a similar spectral characteristic in the dark but undergo different spectral changes upon light absorption, suggesting that the molecular properties of the Opn3 photoproducts differ from those of TMT opsins [5, 26].

### Light-dependent cAMP change in cultured cells expressing vertebrate Opn3

**Fig 2:**
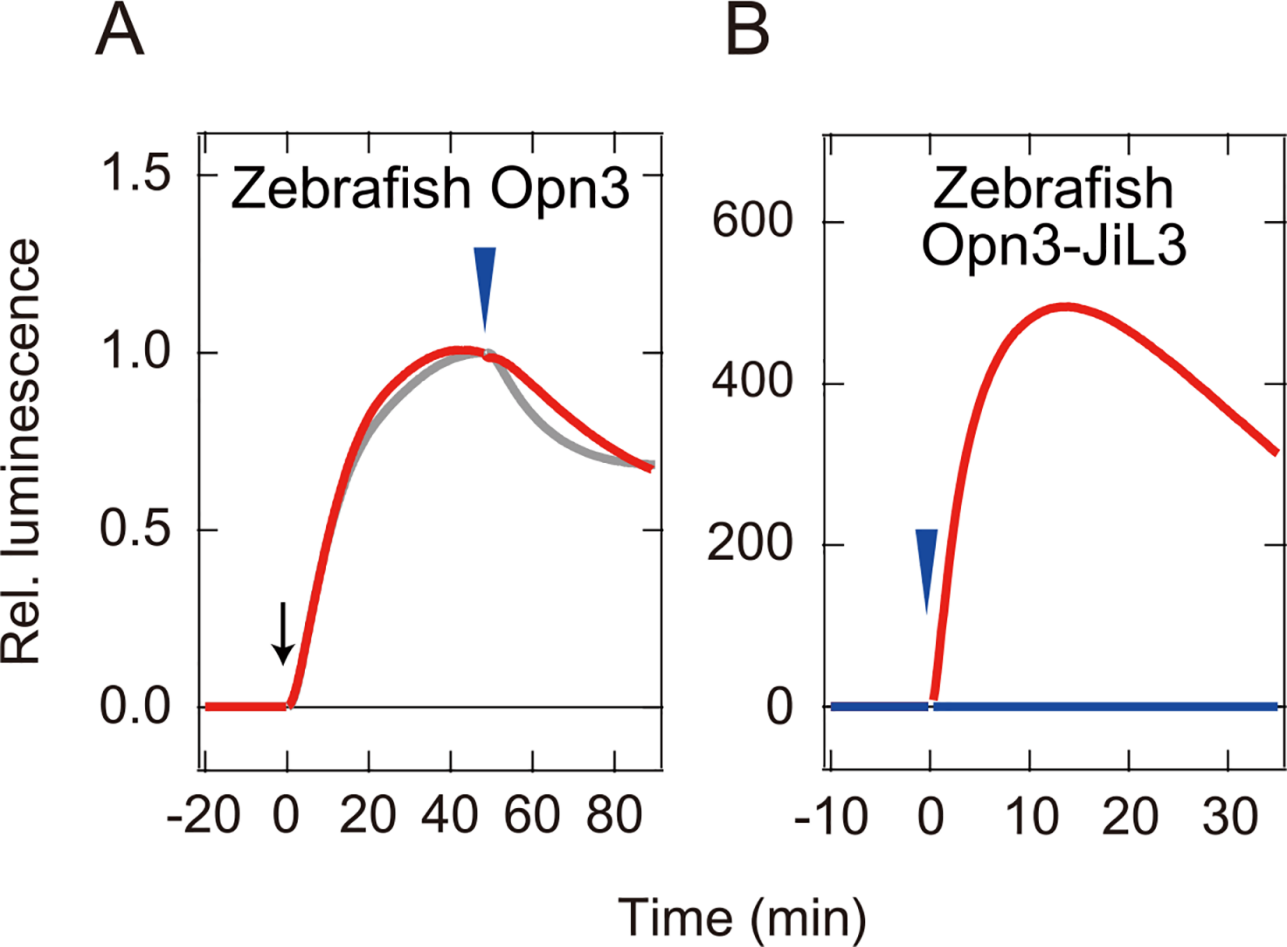
Light-dependent cAMP change in cultured cells expressing zebrafish Opn3. Intracellular cAMP levels in cultured cells were measured as intensity of luminescence signals. (A) Light-dependent cAMP change in zebrafish Opn3 (red curve) and pufferfish TMT opsin (gray curve) after forskolin treatment (black arrow). (B) Light-dependent cAMP change in zebrafish Opn3 (blue curve) and zebrafish Opn3-JiL3 mutant (red curve). Blue arrowheads indicate timing of green light (500 nm) irradiations. The luminescence values were normalized to the average baseline during the 60 seconds immediately preceding forskolin treatment (A) or irradiation (B).

We then used the GloSensor cAMP-dependent luciferase reporter assay to measure the light-dependent changes in intracellular cAMP in cultured cells expressing vertebrate Opn3. We expected to observe a decrease in cAMP upon light stimulation, because the pufferfish TMT opsin decreases cAMP in the cells through light-dependent activation of Gi [5]. However, we did not detect a light-dependent cAMP decrease in cultured cells expressing the zebrafish Opn3 (Fig 2A). Consistent with previous results, clear cAMP decreases were observed in the cells expressing the TMT opsin (Fig 2A). We also did not observe any cAMP decrease in cells expressing human, mouse, chicken, anole, or pufferfish Opn3 (S3 Fig). These results suggested: 1) that vertebrate Opn3 might activate a G protein other than Gi, unlike the vertebrate TMT opsin, or 2) that vertebrate Opn3 may not activate G proteins.

To investigate whether Opn3 has the ability to activate a G protein we analyzed the cAMP response of cultured cells expressing the mutant zebrafish Opn3 (Opn3-JiL3), in which the third intracellular loop was replaced with the third intracellular loop of the jellyfish Gs-coupled opsin [10]. We replaced the native third intracellular loop because this loop is a major determinant of G protein selectivity in opsins [33]. We observed an obvious light-dependent increase of cAMP level in the cells expressing the zebrafish Opn3 mutants (Fig 2B). The results suggest that zebrafish Opn3 may serve as a G protein-coupled photopigment, although the G protein that Opn3 couples remains unknown.

**Fig 3:**
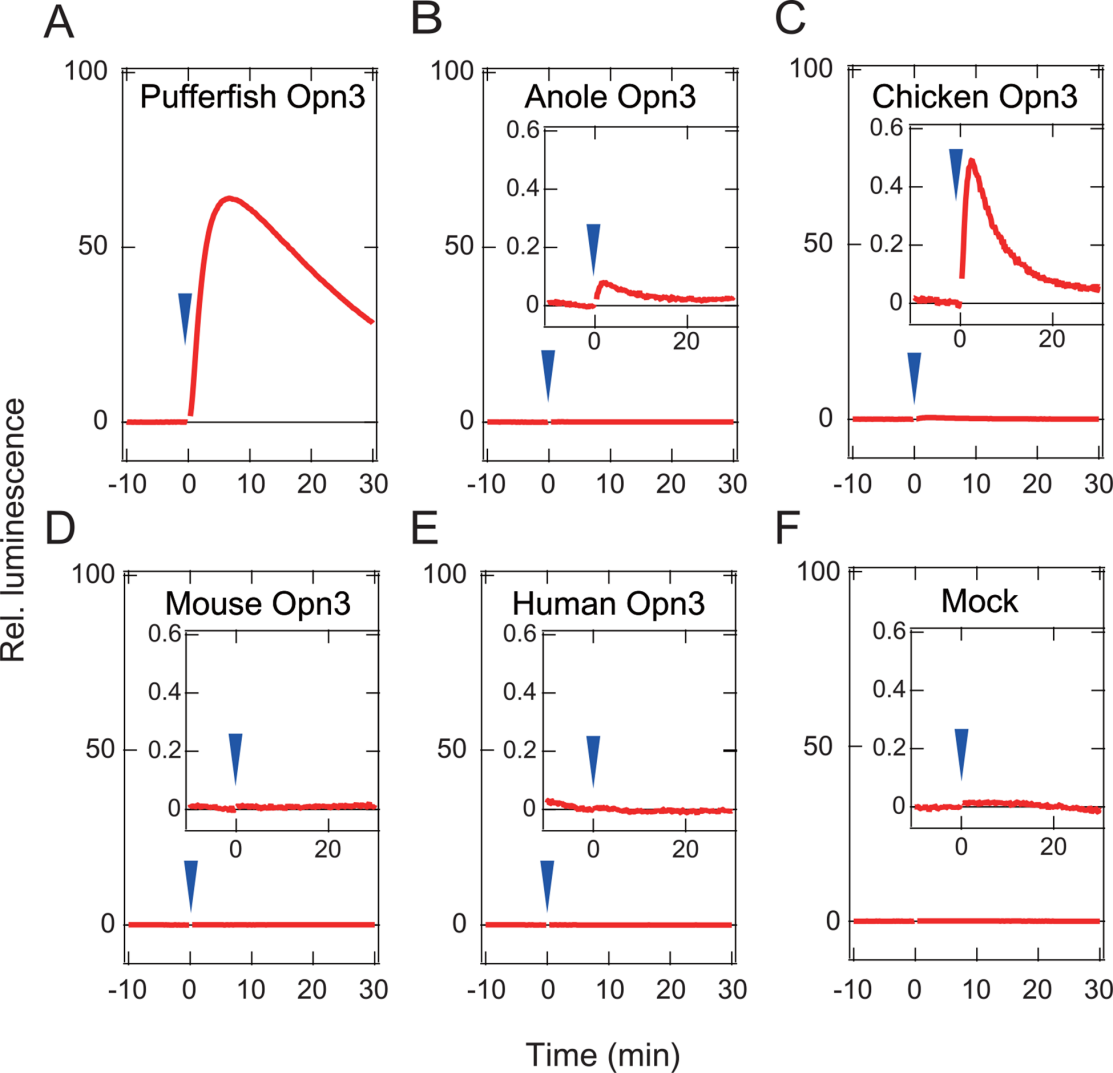
Light-induced intracellular cAMP increases in HEK293 cells expressing Opn3-JiL3 mutants. (A) pufferfish Opn3-JiL3 mutant, (B) anole Opn3-JiL3 mutant, (C) chicken Opn3-JiL3 mutant, (D) mouse Opn3-JiL3 mutant, (E) human Opn3-JiL3 mutant, (F) Mock (cells not transfected with opsins). Arrowheads indicate the timing of green light (500 nm) irradiations. The luminescence values were normalized to the average baseline during the 60 seconds immediately preceding irradiations. Insets in B-F, higher magnified view near zero level.

We also constructed and analyzed similar chimeric mutants of the other Opn3s for which we were unable to measure absorption spectra. Although we could not observe any obvious light-dependent increases in cAMP in cells expressing the mouse and human Opn3-JiL3 mutants, probably due to their low expression level, we observed clear light-dependent responses in the cultured cells expressing the pufferfish, chicken, and anole Opn3 mutants (Fig 3), suggesting that avian and reptilian Opn3s might also serve as a light-sensing proteins, similar to the teleost Opn3s. At the same time, the light-dependent cAMP response observed with these Opn3s prompted us to attempt estimation of their absorption spectra by measuring wavelength-dependent changes in intracellular cAMP.

### Spectral sensitivities of Opn3-based pigments based on wavelength-dependent intracellular responses

**Fig 4:**
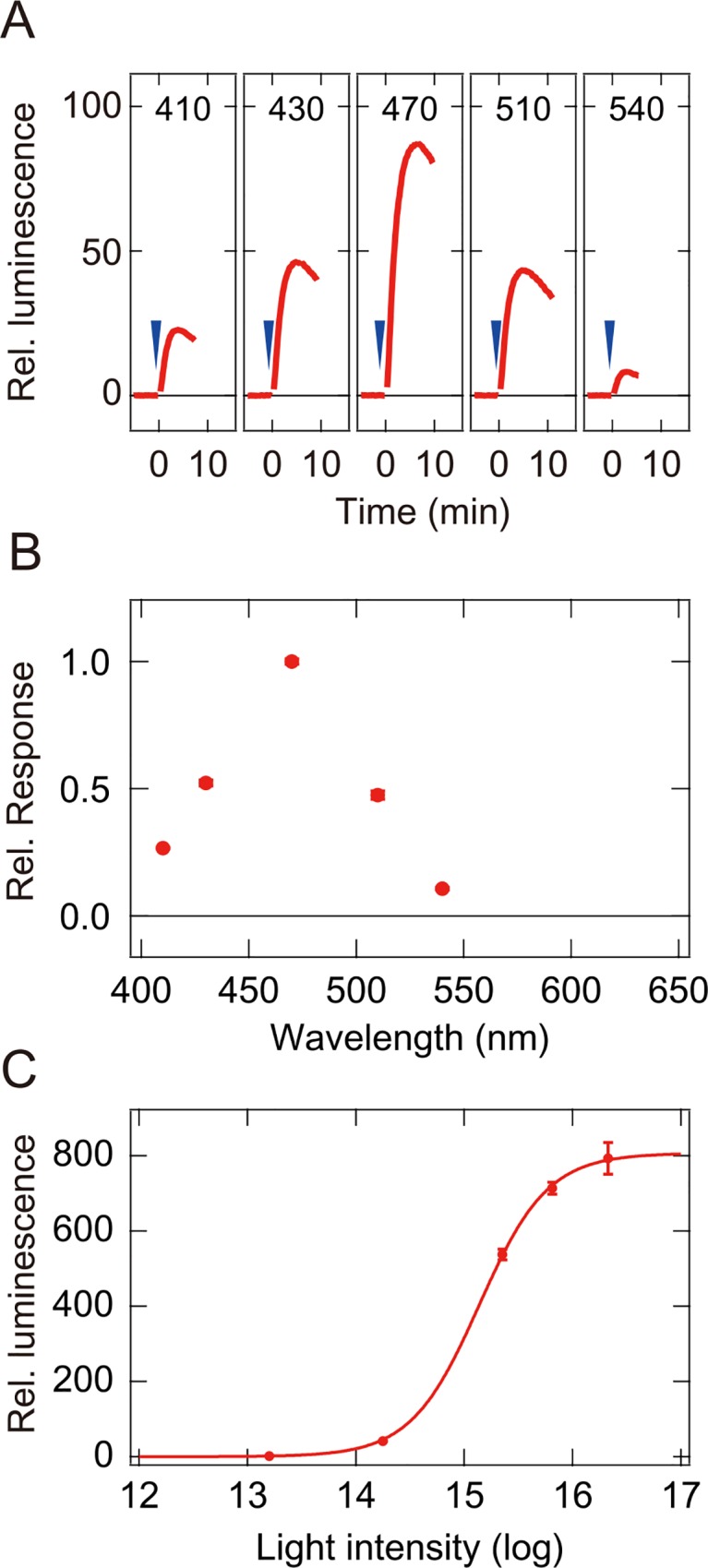
The relative response and the dose-response (light intensity-response) curve of the zebrafish Opn3-JiL3. (A) Response (change in relative luminescence) of cells expressing the zebrafish Opn3-JiL3 upon irradiation with quantally matched light stimuli of five distinct wavelengths (410 nm, 430 nm, 470 nm, 510 nm, and 540 nm). Arrowheads indicate the timing of light irradiation. (B) The relative response of cultured cells expressing the zebrafish Opn3-JiL3. Mean relative responses (red circles) were normalized to the largest mean response (mean response to the 470 nm light stimulus). (C) The dose-response (light intensity-response) for cells expressing the zebrafish Opn3-JiL3 mutant. The intensity-response curve was obtained by fitting a sigmoid function to mean relative responses (mean relative luminescence) to the 500 nm light stimulus at five intensities spanning a 1000-fold range in intensity. The data are presented as the mean ± s.e.m., N = 3.

To estimate the spectral sensitivities of the Opn3-based pigments, we used the GloSensor cAMP-dependent luciferase reporter assay to compare and quantify the relative wavelength-dependent responses of cells expressing the Opn3-JiL3 mutants. We first determined the relative responses at five different wavelengths (410, 430, 470, 510 and 540 nm) for each Opn3-JiL3-expressing cells by quantifying the wavelength-dependent change in intracellular cAMP in the cultured cells upon irradiation with light stimuli of different wavelength composition but equal quantal intensity (Fig 4A and 4B). We then generated a dose-response (light intensity-response) curve for each Opn3-JiL3-expressing cells by quantifying responses to a single stimulus (500 nm) at varying light intensities (Fig 4C). We used the dose-response curves to calculate relative sensitivities at the different wavelength and fit this relative sensitivity data with the rhodopsin template (nomogram curve) to estimate a spectral sensitivity curve [38]. The residual sum of squares was calculated as a function of λmax of nomogram curves (S4 Fig), and the minimum value of residual sum of squares for each Opn3-JiL3 indicates the goodness of fit between the estimated sensitivity curve and sensitivities obtained in the experiments (S4 Fig).

**Fig 5:**
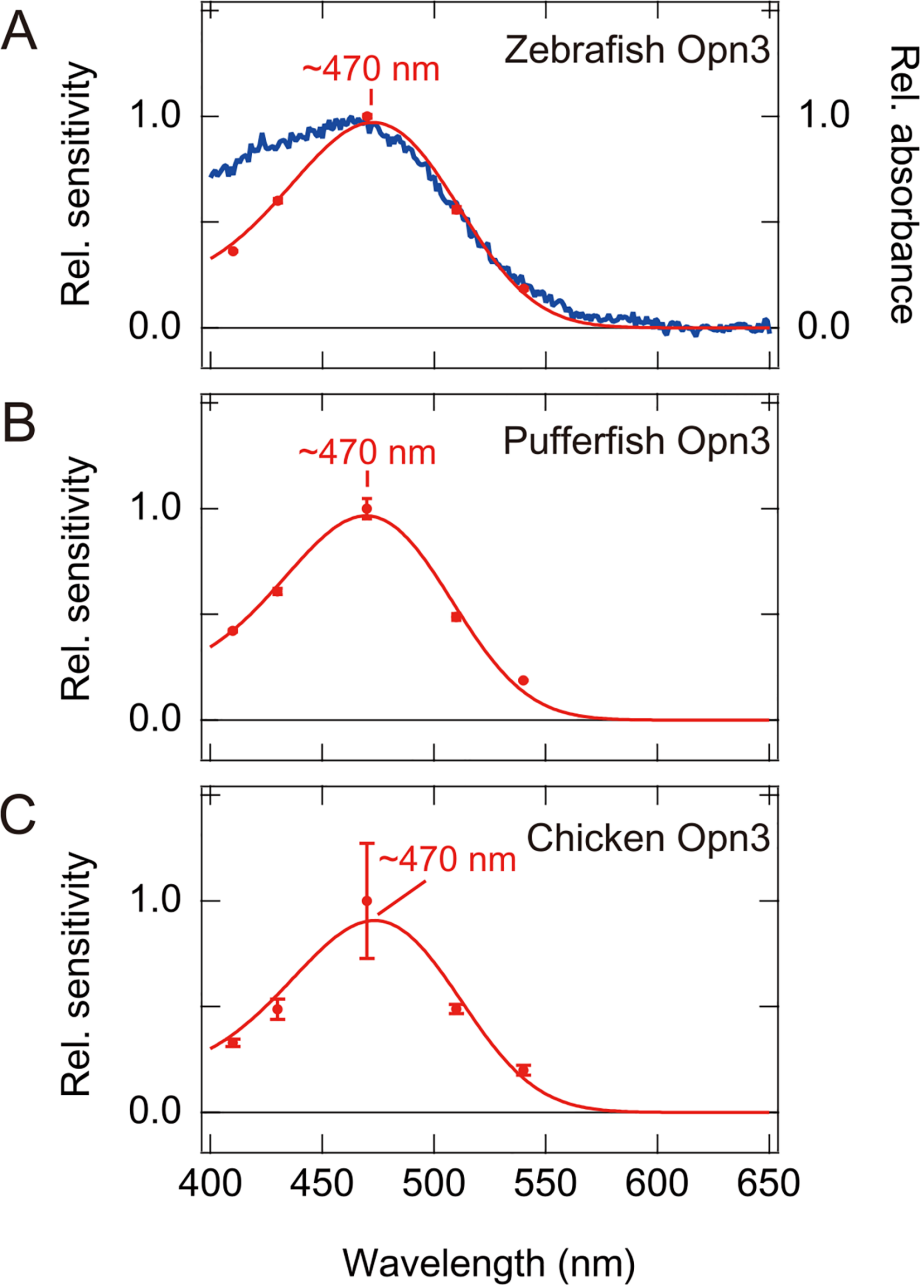
The estimated spectral sensitivity curves of vertebrate Opn3-JiL3 mutants. (A) The spectral sensitivity of zebrafish Opn3-JiL3 (red curve) and the measured absorption spectrum of WT zebrafish Opn3 (blue curve). (B) The spectral sensitivity of pufferfish Opn3-JiL3. (C) The spectral sensitivity of chicken Opn3-JiL3. Red circles represent the mean (n = 3 in A and B, n = 9 in C) relative sensitivities (change in luminescence/cAMP) of cultured cells expressing vertebrate Opn3-Ji L3 mutants at each wavelength of light. Error bars represent standard errors. The λmax of the nomogram was determined for each Opn3 by finding the fit of the relative sensitivity data that resulted in the minimum residual sum of squares, as shown in S4A–S4C Fig. It should be noted that the spectral sensitivity curve of chicken Opn3-JiL3 had a larger standard error at 470 nm than at other wavelengths probably because responses were measured under strong light intensity conditions to obtain clear and reproducible responses. Supplemental information about goodness of fit between the sensitivities experimentally obtained and the fitting curves are shown in S4 Fig.

The spectral sensitivity curve that we calculated for the zebrafish Opn3-JiL3-expressing cells accurately reproduced the absorption spectra curve that we measured for zebrafish Opn3 WT (Fig 5A). The calculated curve matched the absorption spectrum of zebrafish Opn3 well at wavelengths ≥ 470 nm and the wavelengths of the calculated maximum sensitivity (~470 nm) are almost identical to measured absorption maximum (Fig 1). These results show that replacement of the third intracellular loop of opsins (Opn3-JiL3 mutants) can switch the G protein signaling properties, in this case producing a pigment that appears to activate Gs, to generate an intracellular cAMP response while preserving the spectral properties of the WT pigment. To further validate this approach, we tested similar chimeric mutants of the mosquito Opn3 homolog, spider visual opsin Rh1, bovine rhodopsin and native jellyfish opsin. For all opsins, the spectral sensitivity curves predicted using this assay reflected measured spectral sensitivity curves (S5 and S4D–S4G Figs). This gave us confidence that we could accurately estimate the spectral sensitivity of the other vertebrate Opn3s using this method.

We successfully inferred the spectral sensitivity curves of pufferfish and chicken Opn3-JiL3 mutants (Fig 5B and 5C, S4B and S4C Fig). The spectral sensitivity curves of pufferfish and chicken Opn3 indicated that these pigments have maximum sensitivities at ~470 nm, showing that both pufferfish and chicken Opn3 form blue-sensitive pigment. Unfortunately, we were unable to generate a spectral sensitivity curve for the anole Opn3-JiL3 mutant because the light-dependent responses of cells expressing the anole Opn3-JiL3 mutant were too small to reliably discriminate between the responses and baseline (Fig 3B). Likewise, we were unable to estimate the spectral sensitivity of mouse and human Opn3s because we were unable to observe a light-dependent cAMP response in cells expressing these Opn3-JiL3 mutants.

## Discussion

In this report, we succeeded in obtaining an absorption spectrum of zebrafish Opn3 by spectroscopic analysis of the purified pigment (Fig 1) but failed to obtain the spectra for other vertebrate Opn3s, probably due to low expression level in cultured cells. We then performed a Glosensor cAMP-dependent luciferase reporter assay to measure a light-dependent response in cells expressing a zebrafish Opn3 mutant (an Opn3 in which the third intracellular loop was replaced by that of the Gs-coupled jellyfish opsin, Fig 2) and used this assay to quantify the relative response of cells expressing the zebrafish Opn3-JiL3 mutant to estimate its spectral sensitivity (Fig 4). The inferred spectral sensitivity curve closely matched the absorption spectrum that we measured for the WT pigment (Fig 5). We then validated (S5 Fig) and used this assay to demonstrate light-sensitivity in pufferfish, chicken and anole Opn3-JiL3 mutants (Fig 3) and successfully inferred the spectral sensitivity of pufferfish and chicken Opn3s (Fig 5). The results suggest that these vertebrate Opn3s form blue-sensitive pigments (Figs 1 and 5).

Although absorption spectrum of the purified chicken Opn3 was not obtained, we observed a significant light-dependent spectral change by calculating the difference spectrum–the difference in absorbance of the crude extract from chicken Opn3-expressing cultured cells before and after irradiation under alkaline conditions (S6 Fig). It has been reported that irradiation of invertebrate rhodopsins and melanopsin in alkaline conditions (pH 10–11) resulted in bleached photoproducts with deprotonated retinylidene Schiff bases. As a result, the absorbance maximum predicted by the difference spectrum under the alkaline conditions is considered to be identical to that of the purified pigment [39]. Here, we showed that the absorbance maximum predicted from the difference spectrum of the zebrafish Opn3 (~470nm) is almost identical to that of the purified zebrafish Opn3 (S6A Fig). Absorption maximum of chicken Opn3 determined from the difference spectra was ~470 nm, which is almost identical to the maximum sensitivity determined using the GloSensor spectral sensitivity assay. Therefore, although the GloSensor measurements at 470 nm had a large standard error, the difference spectrum supports the inferred relative sensitivity curve of chicken Opn3 and maximum sensitivity at ~470 nm.

The dark absorption spectrum of zebrafish Opn3 is very similar to those of the teleost TMT opsins, sister subgroup members of the vertebrate Opn3s [5, 26] (Fig 1). However, light-induced cAMP changes (a decrease in cAMP), which results from activation of Gi-type proteins and was previously observed in TMT opsin-expressing cells [5], was not observed in cells expressing vertebrate wild-type Opn3 (Fig 2A, S3 Fig). On the other hand, the zebrafish Opn3 mutant, in which the third intracellular loop was replaced with that of jellyfish opsin, resulted in light-dependent increases in cAMP, consistent with activation of Gs, in the cultured cells (Fig 2B). In addition, other vertebrate Opn3-JiL3 mutants (pufferfish, chicken and anole) also produced light-dependent increases in the cAMP level in cultured cells (Fig 3). These findings suggested that the vertebrate Opn3s might activate a different G protein-mediated signal transduction cascade(s) from TMT opsin even though they have similar spectral sensitivities (i.e., they both form blue-sensitive pigments). The difference in the photochemical properties of the Opn3 and TMT opsin photoproducts could support this hypothesis. Therefore, it can be speculated that the vertebrate Opn3 and TMT opsin might have diverged to use different signal transduction cascades. The type(s) of G protein-mediated signal transduction that is activated by vertebrate Opn3s remains an open and important question.

The spectral sensitivity curve of mammalian melanopsin was previously estimated by quantitative measurements of Ca^2+^ elevation in melanopsin-expressing cultured cells [40]. Here, we developed a method for analyzing the spectral sensitivity of opsins, based on quantification of wavelength-dependent cAMP changes in cultured cells expressing opsin-JiL3 mutants. This method is useful for diverse types of opsins, independent of the type of G protein activated by the native pigment, and enables the determination of spectral sensitivity of pigments for which absorption spectra cannot be obtained by spectroscopic analysis. In fact, we used this procedure to accurately estimate the spectral sensitivity curve of zebrafish Opn3 as well as pufferfish and chicken Opn3s, for which we were unable to obtain absorption spectra by spectroscopic analysis of the purified pigments (Fig 5). We have also found that the procedure can be used to accurately estimate spectral sensitivity curves of other opsin-based pigments with different molecular properties, including a bistable pigments with the ability to photoregenerate (Gi/Go-coupled mosquito Opn3 homolog [5] and Gq-coupled spider Rh1 [32]), a bleach-resistant pigment with no obvious photoregeneration ability (Gs-coupled jellyfish opsin [10]) and a bleaching pigment (Gt-coupled bovine rhodopsin) (S5 Fig). These observations suggest that the cell-based spectral sensitivity assay described here, which enables reproducible, quantitative measurements of wavelength-dependent changes in second messenger levels in cultured cells expressing opsin-JiL3 chimeras, may be applicable to diverse types of opsins even if their native properties are unknown and enables the investigation of spectral characteristics with physiological relevance. Until now, thousands of opsin genes have been identified from a variety of animal species but molecular properties, including spectral sensitivities, of a large number of them remain undetermined due to the difficulty of obtaining purified pigments. By enabling the investigation of molecular properties of opsins that had formerly been inaccessible, the current method could facilitate not only the discovery of novel physiological roles of extra-ocular opsins, but also novel spectral tuning mechanisms, and new optogenetic tools for regulating GPCR-signaling.

## Supporting Information

S1 Fig Alignment of amino acid sequences of the third intracellular loops of opsin. Third intracellular loops of opsins (black plane letters) were replaced with that of Gs-coupled jellyfish opsin (red bold letter). The schematic drawing of chimeric mutants is also shown.
(PDF)

S2 Fig Spectra of monochromatic lights. From left to right, spectra of 410 nm, 430 nm, 470 nm, 510 nm, 540nm, 580 nm, 600 nm and 630 nm monochromatic lights are shown.
(PDF)

S3 Fig Light-induced intracellular cAMP decreases in HEK293 cells expressing wild type vertebrate Opn3s. (A) pufferfish Opn3 (B) anole Opn3, (C) chicken Opn3, (D) mouse Opn3, (E) human Opn3, (F) Mock (cells not transfected with opsins). Blue arrowheads and black arrows indicate the timing of green light (500 nm) irradiations and forskolin treatments, respectively. The luminescence values were normalized to the average baseline during the 60 seconds immediately preceding irradiations.
(PDF)

S4 Fig Goodness of fit between experimentally obtained sensitivities and rhodopsin nomogram curves. Residual sum of squares as a function of λmax of various nomograms are shown as an indication of goodness of fit between experimentally obtained sensitivities and the estimated spectral sensitivity curves. (A) zebrafish Opn3-JiL3, (B) pufferfish Opn3-JiL3, (C) chicken Opn3-JiL3, (D) mosquito Opn3-JiL3, (E) spider Rh1-JiL3, (F) jellyfish opsin, (G) bovine rhodopsin-JiL3. The nomogram producing the smallest residual sum of squares value was selected as the best fitting curve for each opsin pigments. Even in the case of mosquito Opn3 (D), which had the largest residual sum of squares, the spectral sensitivity curve is still fit to the absorption spectrum well (S5A Fig), indicating that this approach works well for estimating spectral sensitivity. Therefore, we are confident in the spectral sensitivity curve calculated for chicken Opn3 in spite of the large standard error observed associated with the responses measured at 470 nm (Fig 5C).
(PDF)

S5 Fig Estimation of the spectral sensitivities of various opsin-based pigments. The spectral sensitivity curves (red curves) of cells expressing mosquito Opn3-JiL3 mutant (A), spider Rh1-JiL3 (B), jellyfish opsin WT (C), and bovine rhodopsin-JiL3 mutant (D) with absorption spectra of the respective wild type pigments (blue curves). The spectral sensitivity curves were estimated by fitting a rhodopsin nomogram to mean values of relative sensitivities. It should be noted that these opsins possess different molecular properties of the photoproducts. The photoproduct of mosquito Opn3 is stable, bleach-resistant and reverts to the original dark state upon light absorption, showing the pigments bistable nature and ability to photoregenerate. In contrast, the jellyfish opsin photoproduct is bleach-resistant but does not have the clear photoregeneration ability. The bovine rhodopsin photoproduct is unstable, releases its chromophore and bleaches.
(PDF)

S6 Fig Spectral changes of crude extract containing zebrafish Opn3 and chicken Opn3 during irradiation in alkaline condition. Difference spectra of crude extracts from the cultured cells expressing zebrafish Opn3 (A) and chicken Opn3 (B) at ~ pH 10. The difference absorption spectra were generated by subtracting values obtained before from after irradiation with blue light for 4 min. It should be noted that the absorption maximum of difference spectra at alkaline pH indicates an absorption maximum for the dark spectrum of an opsin-based pigment when photoproduct has lower pKa value for Schiff base protonation than dark state, like invertebrate rhodopsins and melanopsins [18].
(PDF)

## References

[1] Terakita A. The opsins. Genome Biol. 2005;6(3):213 . 15774036 10.1186/gb-2005-6-3-213 PMC1088937

[2] Koyanagi M, Terakita A. Diversity of animal opsin-based pigments and their optogenetic potential. Biochimica et biophysica acta. 2014;1837(5):710–6. 10.1016/j.bbabio.2013.09.003 . 24041647

[3] Terakita A, Nagata T. Functional properties of opsins and their contribution to light-sensing physiology. Zoological science. 2014;31(10):653–9. 10.2108/zs140094 . 25284384

[4] Kuhn H. Light- and GTP-regulated interaction of GTPase and other proteins with bovine photoreceptor membranes. Nature. 1980;283(5747):587–9. . 6101903 10.1038/283587a0

[5] Koyanagi M, Takada E, Nagata T, Tsukamoto H, Terakita A. Homologs of vertebrate Opn3 potentially serve as a light sensor in nonphotoreceptive tissue. Proceedings of the National Academy of Sciences of the United States of America. 2013;110(13):4998–5003. 10.1073/pnas.1219416110 23479626 PMC3612648

[6] Terakita A, Hariyama T, Tsukahara Y, Katsukura Y, Tashiro H. Interaction of GTP-binding protein Gq with photoactivated rhodopsin in the photoreceptor membranes of crayfish. FEBS Lett. 1993;330(2):197–200. . 8365491 10.1016/0014-5793(93)80272-v

[7] Lee YJ, Shah S, Suzuki E, Zars T, O'Day PM, Hyde DR. The Drosophila dgq gene encodes a G alpha protein that mediates phototransduction. Neuron. 1994;13(5):1143–57. . 7946351 10.1016/0896-6273(94)90052-3

[8] Kikkawa S, Tominaga K, Nakagawa M, Iwasa T, Tsuda M. Simple purification and functional reconstitution of octopus photoreceptor Gq, which couples rhodopsin to phospholipase C. Biochemistry. 1996;35(49):15857–64. . 8961950 10.1021/bi961360v

[9] Kojima D, Terakita A, Ishikawa T, Tsukahara Y, Maeda A, Shichida Y. A novel Go-mediated phototransduction cascade in scallop visual cells. J Biol Chem. 1997;272(37):22979–82. 9287291 10.1074/jbc.272.37.22979

[10] Koyanagi M, Takano K, Tsukamoto H, Ohtsu K, Tokunaga F, Terakita A. Jellyfish vision starts with cAMP signaling mediated by opsin-G(s) cascade. Proceedings of the National Academy of Sciences of the United States of America. 2008;105(40):15576–80. Epub 2008/10/04. 0806215105 [pii] 10.1073/pnas.0806215105 18832159 PMC2563118

[11] Yamashita T, Ohuchi H, Tomonari S, Ikeda K, Sakai K, Shichida Y. Opn5 is a UV-sensitive bistable pigment that couples with Gi subtype of G protein. Proceedings of the National Academy of Sciences of the United States of America. 2010;107(51):22084–9. Epub 2010/12/08. 1012498107 [pii] 10.1073/pnas.1012498107 21135214 PMC3009823

[12] Kojima D, Mori S, Torii M, Wada A, Morishita R, Fukada Y. UV-sensitive photoreceptor protein OPN5 in humans and mice. PLoS One. 2011;6(10):e26388 Epub 2011/11/02. 10.1371/journal.pone.0026388 PONE-D-11-10418 [pii]. 22043319 PMC3197025

[13] Hara T, Hara R. Regeneration of squid retinochrome. Nature. 1968;219(5153):450–4. Epub 1968/08/03. . 5668423 10.1038/219450a0

[14] Hao W, Fong HK. The endogenous chromophore of retinal G protein-coupled receptor opsin from the pigment epithelium. J Biol Chem. 1999;274(10):6085–90. Epub 1999/02/26. . 10037690 10.1074/jbc.274.10.6085

[15] Koyanagi M, Terakita A, Kubokawa K, Shichida Y. Amphioxus homologs of Go-coupled rhodopsin and peropsin having 11-cis- and all-trans-retinals as their chromophores. FEBS Lett. 2002;531(3):525–8. . 12435605 10.1016/s0014-5793(02)03616-5

[16] Nagata T, Koyanagi M, Tsukamoto H, Terakita A. Identification and characterization of a protostome homologue of peropsin from a jumping spider. J Comp Physiol A Neuroethol Sens Neural Behav Physiol. 2010;196(1):51–9. Epub 2009/12/05. 10.1007/s00359-009-0493-9 . 19960196

[17] Koyanagi M, Terakita A. Gq-coupled rhodopsin subfamily composed of invertebrate visual pigment and melanopsin. Photochem Photobiol. 2008;84(4):1024–30. Epub 2008/06/03. PHP369 [pii] 10.1111/j.1751-1097.2008.00369.x . 18513236

[18] Koyanagi M, Wada S, Kawano-Yamashita E, Hara Y, Kuraku S, Kosaka S, et al Diversification of non-visual photopigment parapinopsin in spectral sensitivity for diverse pineal functions. BMC biology. 2015;13:73 10.1186/s12915-015-0174-9 26370232 PMC4570685

[19] Davies WI, Tamai TK, Zheng L, Fu JK, Rihel J, Foster RG, et al An extended family of novel vertebrate photopigments is widely expressed and displays a diversity of function. Genome research. 2015;25(11):1666–79. 10.1101/gr.189886.115 26450929 PMC4617963

[20] Arendt D, Tessmar-Raible K, Snyman H, Dorresteijn AW, Wittbrodt J. Ciliary photoreceptors with a vertebrate-type opsin in an invertebrate brain. Science. 2004;306(5697):869–71. . 15514158 10.1126/science.1099955

[21] Blackshaw S, Snyder SH. Encephalopsin: a novel mammalian extraretinal opsin discretely localized in the brain. J Neurosci. 1999;19(10):3681–90. Epub 1999/05/11. . 10234000 10.1523/JNEUROSCI.19-10-03681.1999 PMC6782724

[22] Moutsaki P, Whitmore D, Bellingham J, Sakamoto K, David-Gray ZK, Foster RG. Teleost multiple tissue (tmt) opsin: a candidate photopigment regulating the peripheral clocks of zebrafish? Brain Res Mol Brain Res. 2003;112(1–2):135–45. Epub 2003/04/03. S0169328X03000597 [pii]. . 12670711 10.1016/s0169-328x(03)00059-7

[23] Halford S, Freedman MS, Bellingham J, Inglis SL, Poopalasundaram S, Soni BG, et al Characterization of a novel human opsin gene with wide tissue expression and identification of embedded and flanking genes on chromosome 1q43. Genomics. 2001;72(2):203–8. 10.1006/geno.2001.6469 . 11401433

[24] Kasper G, Taudien S, Staub E, Mennerich D, Rieder M, Hinzmann B, et al Different structural organization of the encephalopsin gene in man and mouse. Gene. 2002;295(1):27–32. . 12242008 10.1016/s0378-1119(02)00799-0

[25] White JH, Chiano M, Wigglesworth M, Geske R, Riley J, White N, et al Identification of a novel asthma susceptibility gene on chromosome 1qter and its functional evaluation. Human molecular genetics. 2008;17(13):1890–903. 10.1093/hmg/ddn087 . 18344558

[26] Sakai K, Yamashita T, Imamoto Y, Shichida Y. Diversity of Active States in TMT Opsins. PLoS One. 2015;10(10):e0141238 10.1371/journal.pone.0141238 26491964 PMC4619619

[27] Fischer RM, Fontinha BM, Kirchmaier S, Steger J, Bloch S, Inoue D, et al Co-expression of VAL- and TMT-opsins uncovers ancient photosensory interneurons and motorneurons in the vertebrate brain. PLoS biology. 2013;11(6):e1001585 10.1371/journal.pbio.1001585 23776409 PMC3679003

[28] Terakita A, Tsukamoto H, Koyanagi M, Sugahara M, Yamashita T, Shichida Y. Expression and comparative characterization of Gq-coupled invertebrate visual pigments and melanopsin. Journal of neurochemistry. 2008;105(3):883–90. 10.1111/j.1471-4159.2007.05184.x . 18088357

[29] Sun L, Kawano-Yamashita E, Nagata T, Tsukamoto H, Furutani Y, Koyanagi M, et al Distribution of mammalian-like melanopsin in cyclostome retinas exhibiting a different extent of visual functions. PLoS One. 2014;9(9):e108209 10.1371/journal.pone.0108209 25251771 PMC4177573

[30] Airan RD, Thompson KR, Fenno LE, Bernstein H, Deisseroth K. Temporally precise in vivo control of intracellular signalling. Nature. 2009;458(7241):1025–9. Epub 2009/03/20. nature07926 [pii] 10.1038/nature07926 . 19295515

[31] Molday RS, MacKenzie D. Monoclonal antibodies to rhodopsin: characterization, cross-reactivity, and application as structural probes. Biochemistry. 1983;22(3):653–60. . 6188482 10.1021/bi00272a020

[32] Nagata T, Koyanagi M, Tsukamoto H, Saeki S, Isono K, Shichida Y, et al Depth perception from image defocus in a jumping spider. Science. 2012;335(6067):469–71. 10.1126/science.1211667 . 22282813

[33] Terakita A, Yamashita T, Nimbari N, Kojima D, Shichida Y. Functional interaction between bovine rhodopsin and G protein transducin. J Biol Chem. 2002;277(1):40–6. . 11606568 10.1074/jbc.M104960200

[34] Koyanagi M, Kawano E, Kinugawa Y, Oishi T, Shichida Y, Tamotsu S, et al Bistable UV pigment in the lamprey pineal. Proceedings of the National Academy of Sciences of the United States of America. 2004;101(17):6687–91. . 15096614 10.1073/pnas.0400819101 PMC404106

[35] Tsukamoto H, Farrens DL. A constitutively activating mutation alters the dynamics and energetics of a key conformational change in a ligand-free G protein-coupled receptor. J Biol Chem. 2013;288(39):28207–16. 10.1074/jbc.M113.472464 23940032 PMC3784730

[36] Terakita A, Hara R, Hara T. Retinal-binding protein as a shuttle for retinal in the rhodopsin- retinochrome system of the squid visual cells. Vision Res. 1989;29(6):639–52. 2626821 10.1016/0042-6989(89)90026-6

[37] Arikawa K, Mizuno S, Kinoshita M, Stavenga DG. Coexpression of two visual pigments in a photoreceptor causes an abnormally broad spectral sensitivity in the eye of the butterfly Papilio xuthus. J Neurosci. 2003;23(11):4527–32. . 12805293 10.1523/JNEUROSCI.23-11-04527.2003 PMC6740815

[38] Govardovskii VI, Fyhrquist N, Reuter T, Kuzmin DG, Donner K. In search of the visual pigment template. Visual neuroscience. 2000;17(4):509–28. . 11016572 10.1017/s0952523800174036

[39] Koyanagi M, Kubokawa K, Tsukamoto H, Shichida Y, Terakita A. Cephalochordate melanopsin: evolutionary linkage between invertebrate visual cells and vertebrate photosensitive retinal ganglion cells. Curr Biol. 2005;15(11):1065–9. . 15936279 10.1016/j.cub.2005.04.063

[40] Bailes HJ, Lucas RJ. Human melanopsin forms a pigment maximally sensitive to blue light (lambdamax approximately 479 nm) supporting activation of G(q/11) and G(i/o) signalling cascades. Proceedings Biological sciences / The Royal Society. 2013;280(1759):20122987 10.1098/rspb.2012.2987 23554393 PMC3619500

