# Supplementary material for: Absorption Characteristics of Vertebrate Non-Visual Opsin, Opn3
Source: PLoS One. 2016 Aug 17;11(8):e0161215. doi: 10.1371/journal.pone.0161215 (PMC4988782; doi:10.1371/journal.pone.0161215)
Supplement: S1 Fig — Third intracellular loops of opsins (black plane letters) were replaced with that of Gs-coupled jellyfish opsin (red bold letter). The schematic drawing of chimeric mutants is also shown. (PDF) [file pone.0161215.s001.pdf]

Third intracellular loop

**Zebrafish Opn3**	198	**PVGVMVYCYG**NILYTVKMLRS----------IQDLQTVQTIKILRYEKKVA**VMFLMMISCF**	248
**Pufferfish Opn3**	196	**PVGIMIYCYG**NILYAVQMIRS----------IQDLQTVQIIKILRYEKKVS**VMFFLMISCF**	246
**Anole Opn3**	219	**PVGIMAYCYG**HILHAIRMLRC----------VEDLQSIQVIKILRYEKKVA**KMCFLMVTTF**	269
**Chicken Opn3**	207	**PVVIMAYCYG**HILYAVRMLRC----------VEDFQTSQVIKLLKYEKKVA**KMCFLMISTF**	257
**Mouse Opn3**	212	**PVGIIAHCYG**HILYSVRMLRC----------VEDLQTIQVIKMLRYEKKVA**KMCFLMAFVF**	262
**Human Opn3**	214	**PLGVIAHCYG**HILYSIRMLRC----------VEDLQTIQVIKILKYEKKLA**KMCFLMIFTF**	264
**Mosquito Opn3**	200	**PLIVIVYSYT**NIIVYMRRNSA-----------------RVGRINRAEQRVT**SMVAVMIVAF**	243
**Spider rhodopsin**	226	**PLFIIIYCYA**FIVMQVAAHEKSLREQAKKMNIKSLRSNEDNKKASAEFRLA**KVAFMTICCW**	286
**Bovine rhodopsin**	215	**PLIVIFFCYG**QLVFTVKEAAA--------------QQQESATTQKAEKEVT**RMVIIMVIAF**	261
**Opsin-JiL3 mutants**		Jellyfish opsin	
**Jellyfish opsin**	184	PVLLMVATYV**LVQGEMKNMRGRAAQ------LFGSESEAALKNIKAEKRHT**RLVFVMILSF	238

S1 Fig Sugihara et al.
