# Supplementary figures and images for: Absorption Characteristics of Vertebrate Non-Visual Opsin, Opn3
Source: PLoS One. 2016 Aug 17;11(8):e0161215. doi: 10.1371/journal.pone.0161215 (PMC4988782; doi:10.1371/journal.pone.0161215)

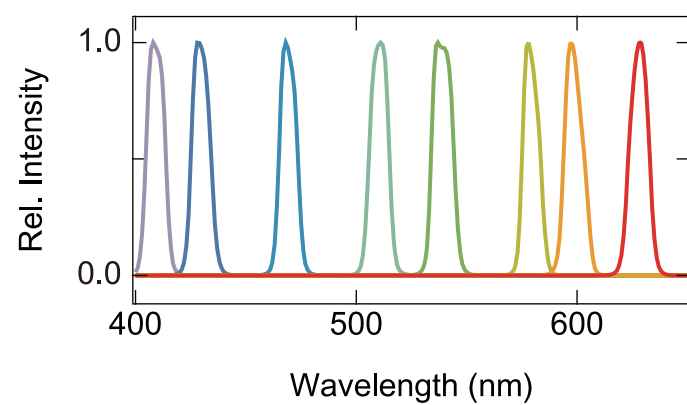

S2 Fig Sugihara et al.

Supplement: S2 Fig — From left to right, spectra of 410 nm, 430 nm, 470 nm, 510 nm, 540nm, 580 nm, 600 nm and 630 nm monochromatic lights are shown. (PDF) [file pone.0161215.s002.pdf]

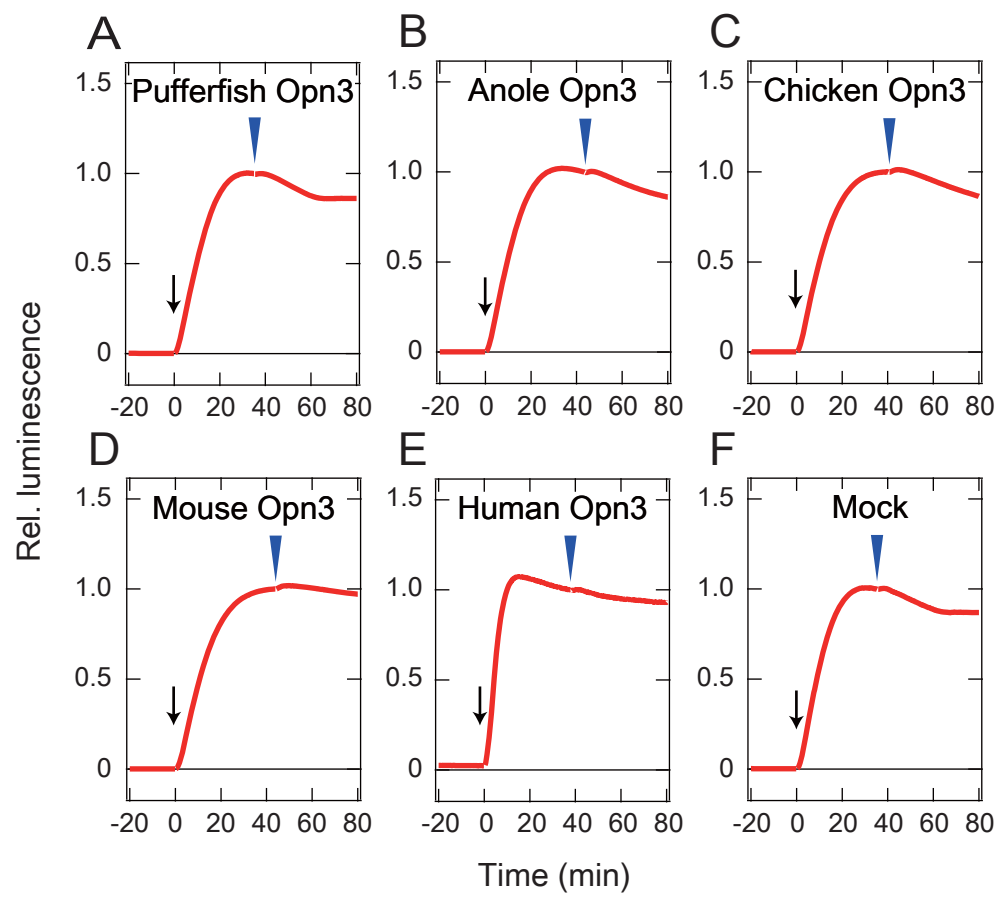

S3 Fig Sugihara et al.

Supplement: S3 Fig — (A) pufferfish Opn3 (B) anole Opn3, (C) chicken Opn3, (D) mouse Opn3, (E) human Opn3, (F) Mock (cells not transfected with opsins). Blue arrowheads and black arrows indicate the timing of green light (500 nm) irradiations and forskolin treatments, respectively. The luminescence values were normalized to the average baseline during the 60 seconds immediately preceding irradiations. (PDF) [file pone.0161215.s003.pdf]

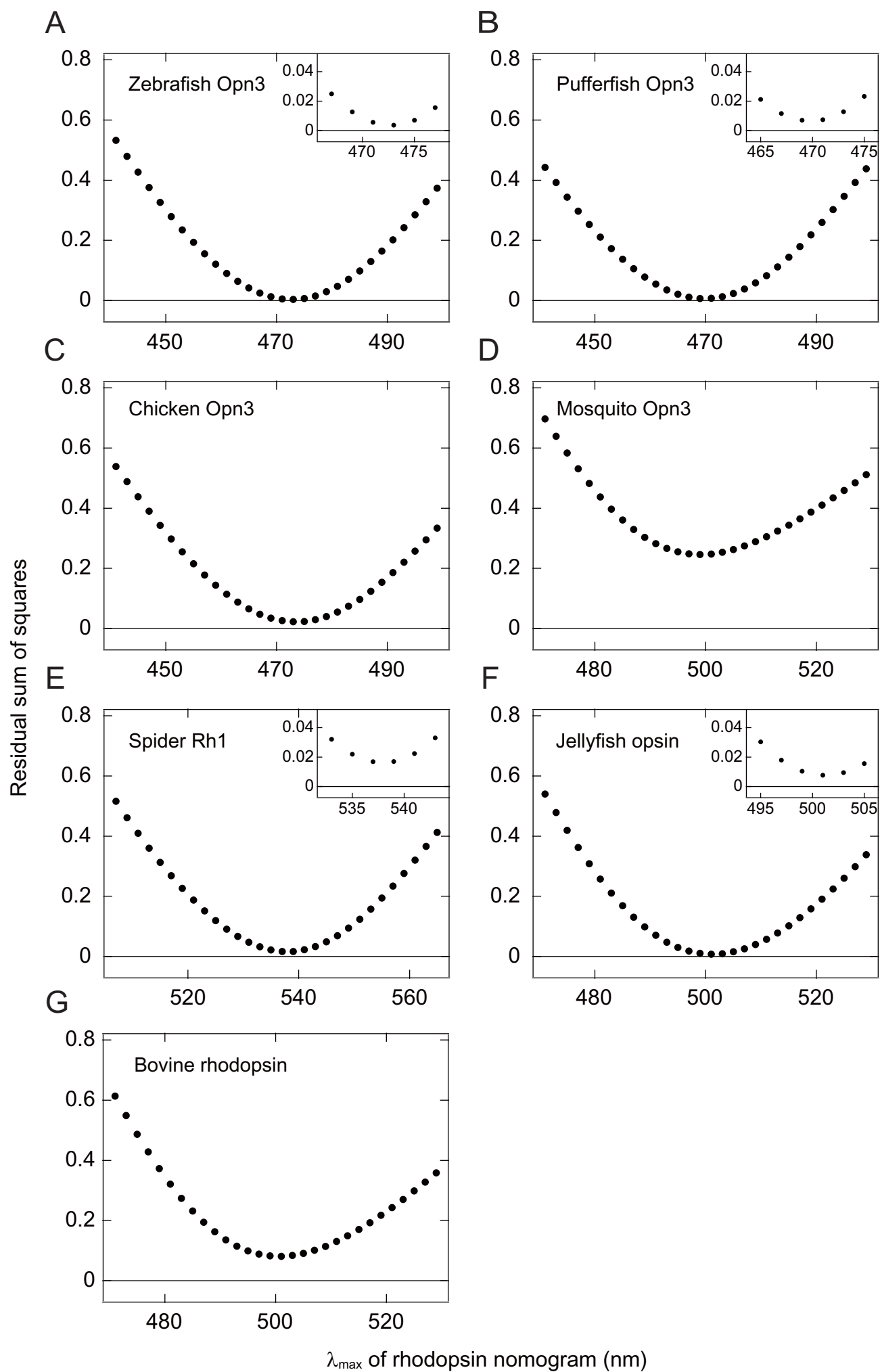

Supplement: S4 Fig — Residual sum of squares as a function of λmax of various nomograms are shown as an indication of goodness of fit between experimentally obtained sensitivities and the estimated spectral sensitivity curves. (A) zebrafish Opn3-JiL3, (B) pufferfish Opn3-JiL3, (C) chicken Opn3-JiL3, (D) mosquito Opn3-JiL3, (E) spider Rh1-JiL3, (F) jellyfish opsin, (G) bovine rhodopsin-JiL3. The nomogram producing the smallest residual sum of squares value was selected as the best fitting curve for each opsin pigments. Even in the case of mosquito Opn3 (D), which had the largest residual sum of squares, the spectral sensitivity curve is still fit to the absorption spectrum well (S5A Fig), indicating that this approach works well for estimating spectral sensitivity. Therefore, we are confident in the spectral sensitivity curve calculated for chicken Opn3 in spite of the large standard error observed associated with the responses measured at 470 nm (Fig 5C). (PDF) [file pone.0161215.s004.pdf]

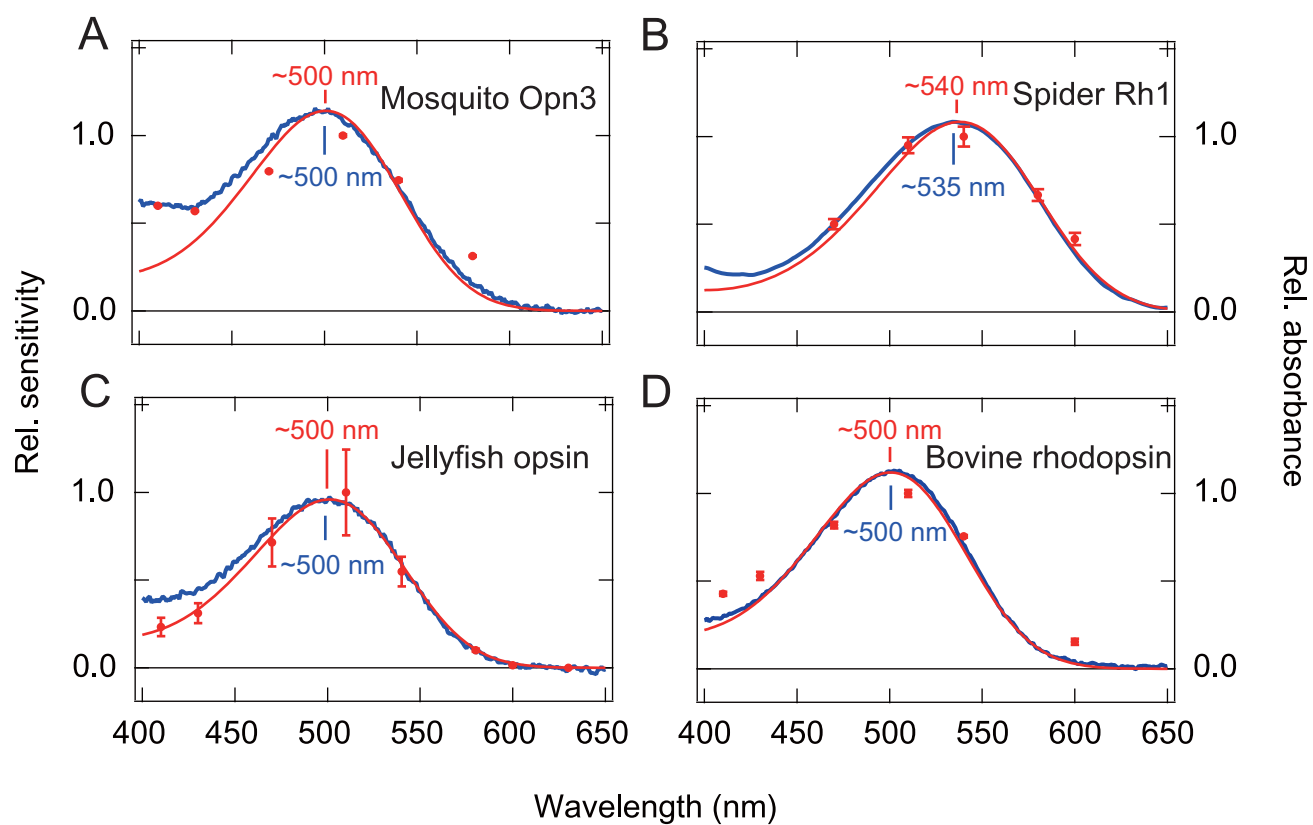

S5 Fig Sugihara et al.

Supplement: S5 Fig — The spectral sensitivity curves (red curves) of cells expressing mosquito Opn3-JiL3 mutant (A), spider Rh1-JiL3 (B), jellyfish opsin WT (C), and bovine rhodopsin-JiL3 mutant (D) with absorption spectra of the respective wild type pigments (blue curves). The spectral sensitivity curves were estimated by fitting a rhodopsin nomogram to mean values of relative sensitivities. It should be noted that these opsins possess different molecular properties of the photoproducts. The photoproduct of mosquito Opn3 is stable, bleach-resistant and reverts to the original dark state upon light absorption, showing the pigments bistable nature and ability to photoregenerate. In contrast, the jellyfish opsin photoproduct is bleach-resistant but does not have the clear photoregeneration ability. The bovine rhodopsin photoproduct is unstable, releases its chromophore and bleaches. (PDF) [file pone.0161215.s005.pdf]

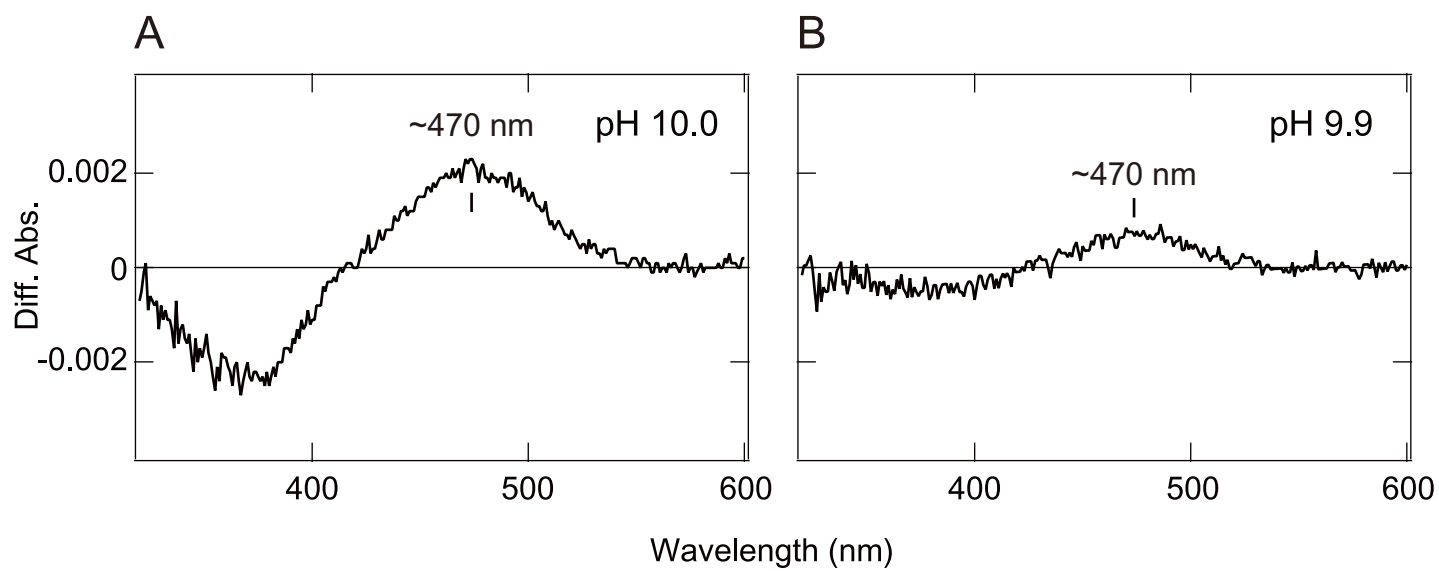

S6 Fig Sugihara et al.

Supplement: S6 Fig — Difference spectra of crude extracts from the cultured cells expressing zebrafish Opn3 (A) and chicken Opn3 (B) at ~ pH 10. The difference absorption spectra were generated by subtracting values obtained before from after irradiation with blue light for 4 min. It should be noted that the absorption maximum of difference spectra at alkaline pH indicates an absorption maximum for the dark spectrum of an opsin-based pigment when photoproduct has lower pKa value for Schiff base protonation than dark state, like invertebrate rhodopsins and melanopsins [18]. (PDF) [file pone.0161215.s006.pdf]
